# Joint analysis of proteome, transcriptome, and multi-trait analysis to identify novel Parkinson’s disease risk genes

**DOI:** 10.18632/aging.205444

**Published:** 2024-01-17

**Authors:** Jing-Jing Shi, Cheng-Yuan Mao, Ya-Zhou Guo, Yu Fan, Xiao-Yan Hao, Shuang-Jie Li, Jie Tian, Zheng-Wei Hu, Meng-Jie Li, Jia-Di Li, Dong-Rui Ma, Meng-Nan Guo, Chun-Yan Zuo, Yuan-Yuan Liang, Yu-Ming Xu, Jian Yang, Chang-He Shi

**Affiliations:** 1 Department of Neurology, The First Affiliated Hospital of Zhengzhou University, Zhengzhou University, Zhengzhou 450000, Henan, China; 2 School of Life Sciences, Westlake University, Hangzhou 310024, Zhejiang, China; 3 Zhengzhou Railway Vocational and Technical College, Zhengzhou 450000, Henan, China; 4 NHC Key Laboratory of Prevention and Treatment of Cerebrovascular Diseases, The First Affiliated Hospital of Zhengzhou University, Zhengzhou University, Zhengzhou 450000, Henan, China; 5 Henan Key Laboratory of Cerebrovascular Diseases, The First Affiliated Hospital of Zhengzhou University, Zhengzhou University, Zhengzhou 450000, Henan, China; 6 Institute of Neuroscience, Zhengzhou University, Zhengzhou 450000, Henan, China; 7 Westlake Laboratory of Life Sciences and Biomedicine, Hangzhou 310024, Zhejiang, China

**Keywords:** Parkinson’s disease, transcriptome-wide association study, proteome-wide association studies, fine-mapping, MTAG

## Abstract

Genome-wide association studies (GWAS) have identified multiple risk variants for Parkinson’s disease (PD). Nevertheless, how the risk variants confer the risk of PD remains largely unknown. We conducted a proteome-wide association study (PWAS) and summary-data-based mendelian randomization (SMR) analysis by integrating PD GWAS with proteome and protein quantitative trait loci (pQTL) data from human brain, plasma and CSF. We also performed a large transcriptome-wide association study (TWAS) and Fine-mapping of causal gene sets (FOCUS), leveraging joint-tissue imputation (JTI) prediction models of 22 tissues to identify and prioritize putatively causal genes. We further conducted PWAS, SMR, TWAS, and FOCUS using a multi-trait analysis of GWAS (MTAG) to identify additional PD risk genes to boost statistical power. In this large-scale study, we identified 16 genes whose genetically regulated protein abundance levels were associated with Parkinson’s disease risk. We undertook a large-scale analysis of PD and correlated traits, through TWAS and FOCUS studies, and discovered 26 casual genes related to PD that had not been reported in previous TWAS. 5 genes (*CD38*, *GPNMB*, *RAB29*, *TMEM175*, *TTC19*) showed significant associations with PD at both the proteome-wide and transcriptome-wide levels. Our study provides new insights into the etiology and underlying genetic architecture of PD.

## INTRODUCTION

Parkinson’s disease (PD) is the second most common neurodegenerative disorder characterized by resting tremor, muscle rigidity, bradykinesia and postural instability [1, 2]. PD is one of the most common alpha-synucleinopathy disorders caused by the abnormal accumulation of alpha-synuclein polymer in pathological inclusion bodies [3]. Lewy body dementia (LBD) [4], idiopathic rapid eye movement sleep behavior disorder (iRBD) [5], and multiple system atrophy are also alpha-synucleinopathy disorders in which some clinical features overlap [6].

Genome-wide association analysis (GWAS) is the application of millions of single nucleotide polymorphisms (SNPs) across the genome as molecular genetic markers for conducting comparative or correlation analyses at the genome-wide level. GWAS involves comparing genetic variations to identify genes that influence complex traits [7, 8]. Nevertheless, these investigations are constrained to delineating the PD risk within a genomic region that includes multiple candidate genes. The specific genes responsible for the pathology at each region and the mechanisms through which they contribute to the risk of PD remain elusive. The majority of risk variants identified in GWASs are situated within noncoding regions, implying that these variants may impact disease susceptibility through the modulation of gene expression [9–12]. Recently, proteome-wide/transcriptome-wide association study (PWAS/TWAS), emerging as a new method, has been designed to explore the association between gene expression and disease across different tissues [13–17]. PWAS/TWAS are powerful approaches that integrate the gene expression reference panel (protein quantitative trait loci [pQTL] or expression quantitative trait loci [eQTL] cohort) and genome-wide associations from large scale GWAS to identify genes with cis-regulated expression associated with complex diseases [18–20]. Multi-trait analysis of GWAS (MTAG) can jointly analyze multiple traits, thereby enhancing statistical power for the detection of genetic associations related to each trait [21–23].

Until recently, three TWAS studies in PD had been performed and identified a series of PD risk genes, while there are still some limitations that should be noted [24–27] (Supplementary Table 1). Firstly, given the pivotal involvement of proteins in the majority of biological processes and the non-linear correlation between messenger ribonucleic acid (mRNA) and protein levels (with an approximate overlap of 35% between eQTL and pQTL) [28], it is crucial to explore whether the reported risk variants have biological impact on PD through the modulation of protein abundance, in which was not investigated in the previous studies. Secondly, the pathological feature of PD is alpha-synucleinopathy, which impacts all levels of the brain–gut axis, including the central nervous system (CNS), autonomic nervous system, and enteric nervous systems. There exists a hypothesis positing the spread of the pathological process from the gastrointestinal tract to the brain [29, 30], while the existing studies did not systematically evaluate tissues besides the CNS, especially in the digestive system. Thirdly, the current investigations predominantly depended on prior PD GWAS datasets characterized by insufficient case and control cohorts for association analyses.

In this study, we use the largest PD GWAS data including 33,674 cases and 449,056 controls. We performed PWAS and SMR [31] by integrating genome-wide associations PD GWAS with human brain and plasma proteomes and pQTL data from human brain, plasma, and CSF. We also performed TWAS and fine-mapping (FOCUS), leveraging joint-tissue imputation (JTI) prediction models of 22 tissues to identify and prioritize putatively causal genes. JIT greatly improves statistical power, replication and causal mapping rates compared with existing models [32]. FOCUS has higher sensitivity in identifying causal genes relative to colocalization (COLOC) [33, 34]. We also conducted PWAS, SMR, TWAS and FOCUS using the results of MTAG to identify additional PD risk genes to boost statistical power.

## RESULTS

### Multi-trait analysis of GWAS for PD, LBD, and RBD resulted in gains equivalent to increasing the original sample, which enhancing the statistical power

**Figure 1:**
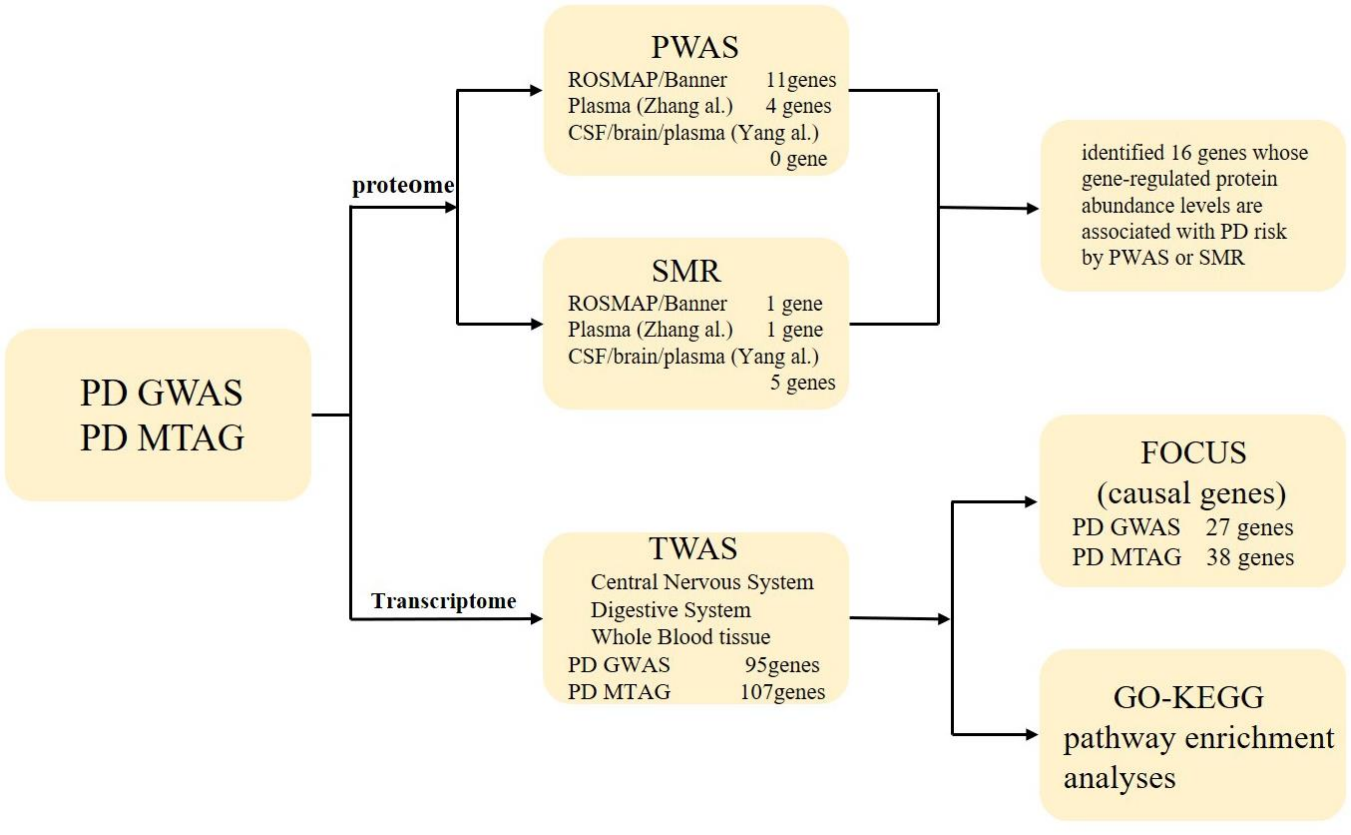
**The workflow of the study.** TWAS, transcriptome-wide association study; PWAS, proteome-wide association study; FOCUS, Fine-mapping of causal gene sets; SMR, summary-data-based mendelian randomization.

Figure 1 summarizes all the analysis methods and procedures applied in this study. We performed multi-trait analysis for PD, LBD, and RBD GWAS using MTAG (Supplementary Table 2 and Supplementary Figure 1). “Lead SNPs” refers to nearly independent genome-wide significant SNPs with a p-value less than 5e^-8^ and low linkage disequilibrium (LD) between SNP loci (r^2^ < 0.1). Lead SNPs are considered to have the strongest association with the studied trait or disease. From single to multi-trait analysis, the number of lead SNPs increased from 32 to 33 for PD, from 5 to 17 for LBD, and from 10 to 10 for RBD. The gain in mean power of each trait MTAG relative to the GWAS results was assessed by the increase in the mean χ2 statistic. We estimated the required GWAS sample size to achieve an equivalent increase in the χ2 statistic. This study found that MTAG analysis for PD, LBD, and RBD resulted in gains equivalent to increasing the original GWAS sample to 504,827 for PD, 89,741 for LBD, and 46,816 for RBD, respectively.

### PWAS identified 15 genes associated with PD risk by regulating protein abundance levels in both the brain and plasma

**Figure 2:**
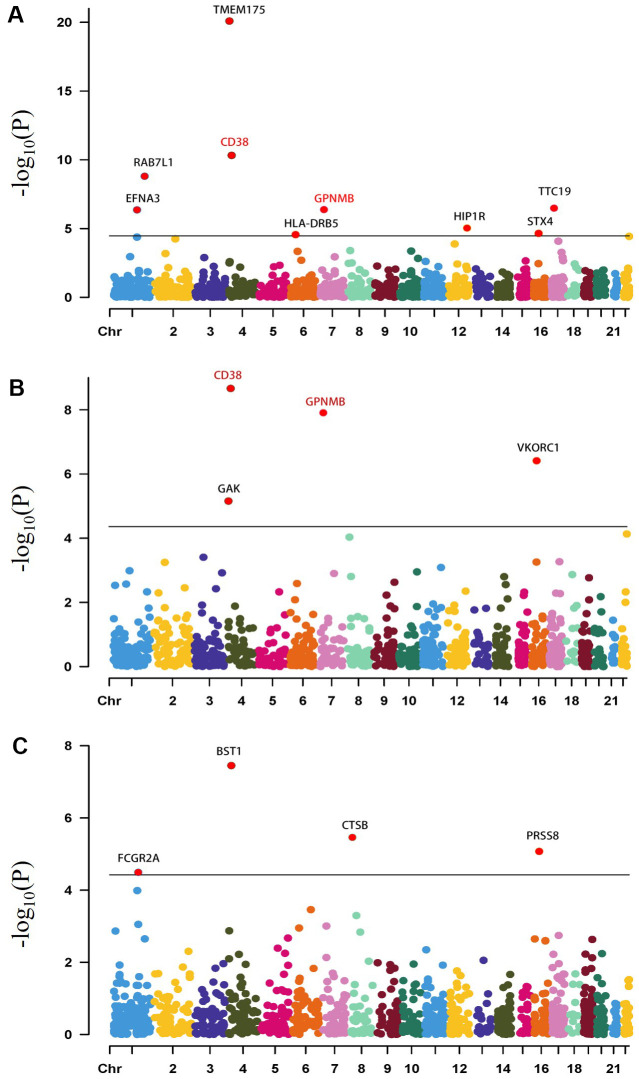
**Manhattan plots for the PD PWASs in the human brain and plasma proteomes.** Manhattan plot for the PWAS integrating the PD GWAS with the ROSMAP proteomes (n= 376) (**A**), Banner proteomes (n= 152) (**B**), plasma proteomes (n= 152) (**C**). Each dot on the x-axis represents a gene, and the association strength on the y-axis represents the -log_10_(p) of PWAS. Proteome-wide significance level was set at p < 4.36×10^-5^ (adjusted by Bonferroni multiple testing correction method) for the Banner dataset. Proteome-wide significance level in the ROSMAP dataset was set at p < 3.39×10^-5^ (adjusted by Bonferroni multiple testing correction method). Proteome-wide significance level in the ROSMAP dataset was set at p < 3.71×10^-5^ (adjusted by Bonferroni multiple testing correction method). Genes that were proteome-wide significant (CD38, GPNMB) in both brain proteomes are shown in red. Chr, chromosome.

We firstly performed PWAS by the ROSMAP (Table 1 and Figure 2A, and Supplementary Table 3) and Banner dataset (Table 1 and Figure 2B, and Supplementary Table 4) and identified 11 proteome-wide significant genes *CD38*, *EFNA3, GAK*, *GPNMB*, *HIP1R*, *HLA*-*DRB5*, *RAB29*, *STX4*, *TMEM175*, *TTC19* and *VKORC1* for PD (adjusted for Bonferroni correction). While using the plasma protein weights, we found four proteome-wide significant genes *FCGR2A*, *BST1*, *CTSB*, and *PRSS8* are associated with PD (Table 1 and Figure 2C and Supplementary Table 5). We did not identify any proteome-wide significant gene for PD in brain, CSF and plasma dataset from a recent proteomics study (Supplementary Tables 6, 7). In summary, by integrating the proteome datasets from different sources and the large-scale PD GWAS, we identified 15 relevant genes associated with PD risk by regulating protein abundance levels in both the brain and plasma. We identified 14 genes (*BST1*, *CD38*, *CTSB*, *EFNA3*, *GAK*, *GPNMB*, *HLA-DRB5*, *HIP1R*, *PRSS8*, *RAB29*, *STX4*, *TMEM175*, *TTC19* and *VKORC1*) associated with the risk of PD using PD MTAG (Supplementary Table 8).

**Table 1:** PWAS and SMR identified PD-associated genes of PD GWAS.

**Gene**	**Chr**	**PWAS**							**SMR**								**TWAS significant**
		**ROSMAP**		**Banner**		**Plasma**			**ROSMAP**		**BRAIN(Yang al)**		**CSF(Yang al)**		**Plasma**		
		**Zscore**	**P-value^a^**	**Zscore**	**P-value^b^**	**Zscore**	**P-value^c^**		**p_SMR**	**p_HEIDI**	**p_SMR**	**p_HEIDI**	**p_SMR**	**p_HEIDI**	**p_SMR**	**p_HEIDI**	
**RAB29**	1	6.04	1.57E-09	-	-	-	-		-	-	-	-	-	-	-	-	Suggestive
EFNA3	1	-5.05	4.39E-07	-	-	-	-		-	-	-	-	-	-	-	-	-
FCGR2A	1	-	-	-	-	4.16	3.22E-05		-	-	8.60E-05	6.85E-01	-	-	-	-	-
FCGR2B	1	-	-	-	-	-	-		-	-	-	-	4.91E-05	5.13E-01	-	-	-
**BST1**	4	-	-	-	-	5.51	3.55E-08		-	-	-	-	-	-	-	-	Suggestive
**CD38**	4	-6.58	4.77E-11	-5.99	2.16E-09	-	-		9.90E-07	7.31E-01	-	-	4.78E-08	7.93E-01	5.85E-01	5.47E-01	Suggestive
**GAK**	4	-	-	-4.49	6.99E-06	-	-		-	-	-	-	-	-	-	-	Suggestive
**TMEM175**	4	-9.36	8.14E-21	-	-	-	-		-	-	-	-	-	-	-	-	Suggestive
HLA-DRB5	6	-4.19	2.78E-05	-	-	-	-		-	-	-	-	-	-	-	-	-
**GPNMB**	7	5.06	4.17E-07	5.69	1.24E-08	-	-		-	-	2.14E-06	4.68E-01	-	-	-	-	Suggestive
**CTSB**	8	-	-	-	-	-4.64	3.46E-06		-	-	-	-	8.02E-05	2.00E-02	-	-	Suggestive
**HIP1R**	12	-4.44	9.21E-06	-	-	-	-		-	-	-	-	-	-	-	-	Suggestive
**VKORC1**	16	-	-	5.08	3.87E-07	-	-		-	-	-	-	-	-	-	-	Suggestive
PRSS8	16	-	-	-	-	-4.45	8.47E-06		-	-	-	-	-	-	-	-	-
**STX4**	16	4.24	2.25E-05	-	-	-	-		-	-	-	-	-	-	-	-	Suggestive
**TTC19**	17	-5.11	3.29E-07	-	-	-	-		-	-	-	-	-	-	-	-	Suggestive

^a^Proteome-wide significance level in the ROSMAP dataset was set at p<3.39E-5 (adjusted by Bonferroni multiple testing correction method).

^b^Proteome-wide significance level in the Banner dataset was set at p<4.36E-5 (adjusted by Bonferroni multiple testing correction method).

^c^Proteome-wide significance level in the Plasma dataset was set at p<3.78E-5 (adjusted by Bonferroni multiple testing correction method).

The genes in bold are the ones that are significant in both the proteome-wide and transcriptome-wide levels.

### SMR reveals putative causal genes associated with PD using brain, plasma and CSF pQTL

In order to further assess whether the association between genetic variants and PD is mediated through cis-regulation of protein abundance, we conducted SMR using the same discovery dataset (ROSMAP/Banner/plasma/CSF/brain). Thus, evidence suggests that the causal effects of five genes (*CD38*, *GPNMB*, *CTSB*, *FCGR2A* and *FCGR2B*) in PD are consistent through the SMR/HEIDI (heterogeneity in dependent instruments) using PD GWAS data (Table 1 and Supplementary Figure 2). We also used PD MTAG data for SMR analysis to identify putative causal genes (Supplementary Table 8). There is evidence indicating a causal relationship between 4 genes (*CD38, GPNMB, CTSB,* and *FCGR2B)* and PD by SMR/HEIDI using PD MTAG data. To summarize, we have identified a total of 16 candidate genes that show associations with PD using brain, plasma and CSF pQTL by PWAS and SMR analysis.

### TWAS and FOCUS identifies candidate genes associated with PD risk in gene expression levels using single and multi-trait analysis GWAS data

In different evaluated tissues, the performance of the predictive model JTI reaches a minimum correlation of 0.01 (indicating a correlation of at least 10% between predicted and measured expression) across a range of quantities, varying from 6,542 to 12,827 (Supplementary Table 9). Utilizing these TWAS models, we identified significant associations between the expression of 95 genes and PD in those 22 tissues. The complete TWAS results for PD in the 22 tissues are included in Supplementary Table 10. Among these 95 genes, 67 are previously unreported genes associated with PD risk identified through TWAS studies (Supplementary Table 11), while there are 28 genes that have been reported in previous PD TWAS studies (Supplementary Table 12). In addition, to illustrate whether genes in the digestive system may influence PD risk, we leveraged seven tissues-specific gene expression prediction models related to digestive system and identified 59 genes associated with the risk of PD. These 11 genes *AC104113*.*1*, *AC116348*.*3*, *CDKL2*, *CR936218*.*2*, *EFNA3*, *ERCC8*, *GBA*, *KANSL1*, *LZTS3*, *PHKG2*, and *TMEM163* have only been shown to be associated with PD in the digestive system (Supplementary Figure 3A). In another separate TWAS analysis specifically focused on whole blood tissue, 26 genes significantly associated with PD were identified. By comparing the results of the three systems, it was found that they had 19 genes in common (Supplementary Figure 3A). Causal genes are based on a gene set with 90% credible by FOCUS, we found that 27 out of 95 were likely causal genes for PD risk (Supplementary Table 13). The 14 of the 27 putative causal genes were not previously identified in prior TWAS studies (Supplementary Table 14). For the genomic locus 3:181511166-3:183769683, *FAM189B* had a posterior probability of 0.948 in the brain cerebellum, and *EFNA3* had a posterior probability of 0.938 in the stomach. For the genomic locus 3:181511166-3:183769683, *MCCC1-AS1* had a posterior probability greater than 0.9 in the brain frontal cortex BA9 and three other brain tissues. And for the genomic locus 4:15147446-4:15926136, *FAM200B* had a posterior probability greater than 0.9 in the cerebellar brain hemisphere. For the genomic loc 4:77130707-4:79093979, *CDKL2* had a posterior probability greater than 0.9 in the stomach. For the genomic locus 5:58524622-5:60935451, *NDUFAF2* had a posterior probability greater than 0.9 in the brain cortex, hippocampus, nucleus accumbent basal ganglia, substantial nigra. For the genomic loc 7:22508611-7:23469560, *KLHL7-DT* had a posterior probability of 0.919 in the brain cerebellum. For the genomic loc 16:29036613-16:31382470, *FBXL19* had a posterior probability of 0.966 in the brain substantia nigra. For the genomic loc 17:15020965-17:16411522, *TTC19* had the posterior probability of 0.924 in the whole blood. For the genomic loc 17:43056905-17:45876022, *MAPT-IT1, KANSL1-AS1, CR936218.1, CR936218.2* had the posterior probability greater than 0.9 in the brain tissues or digestive system.

The multi-trait approach could overall identify loci associated with PD, and our study revealed a significant association between the expression levels of 104 genes and PD using the PD MTAG database in those 22 tissues (Supplementary Tables 15–17). Using the PD MTAG results, we found that 38 of the 104 were likely to be causal genes for PD risk (Supplementary Table 18). The 22 of the 38 putative causal genes were not previously reported in earlier TWAS (Supplementary Table 19). Combining the results of PD and PD MTAG, we identified 26 new putative causal genes that were not previously reported in previous TWAS studies (Table 2 and Supplementary Figure 4).

**Table 2:** Twenty-six identified gene has not been reported to be associated with PD in previous TWAS studies.

**REGION**	**GENE**	**Type**	**TISSUE**	**PD GWAS**			**PDMTAG**		
				**TWAS-P**	**TWAS-Z**	**FOCUS-pip**	**TWAS-P**	**TWAS-Z**	**FOCUS-pip**
1q21.3	EFNA3^b^	protein	Stomach	-	-	-	8.28E-08	4.85	0.938
1q22	FAM189B^b^	protein	Brain_Cerebellum	-	-	-	1.69E-08	5.24	0.948
	GBA^ab^	protein	Spleen	1.29E-08	6.13	1	1.37E-10	6.95	1
2q24.3	STK39^ab^	protein	Brain_Caudate_basal_ganglia	1.13E-07	-5.09	0.98	1.51E-07	-5.05	0.976
		protein	Brain_Nucleus_accumbens_basal_ganglia	1.98E-07	-4.82	0.928	3.27E-07	-4.77	0.911
		protein	Brain_Putamen_basal_ganglia	2.91E-07	-4.94	0.958	2.95E-07	-4.93	0.958
3q27.1	LAMP3^b^	protein	Brain_Putamen_basal_ganglia	-	-	-	8.94E-09	-5	0.914
4p15.32	FAM200B^ab^	protein	Brain_Cerebellar_Hemisphere	2.06E-09	6.06	1	2.65E-08	5.64	0.998
4p16.3	DGKQ^ab^	protein	Brain_Cerebellar_Hemisphere	-	-	-	6.42E-24	-10.4	1
		protein	Brain_Amygdala	4.68E-08	-5.21	1	-	-	-
		protein	Brain_Hippocampus	4.68E-08	-5.21	0.997	-	-	-
		protein	Brain_Cerebellum	-	-	-	5.60E-23	-10	1
		protein	Brain_Substantia_nigra	6.10E-12	-5.21	1	-	-	-
		protein	Brain_Spinal_cord_cervical_c-1	4.30E-12	-5.25	1	9.40E-09	-5.48	1
		protein	Colon_Sigmoid	9.37E-13	-7.26	1	2.37E-19	-8.59	1
		protein	Colon_Transverse	1.09E-11	-7.31	1	2.71E-17	-7.92	1
		protein	Liver	8.32E-13	-7.18	1	9.35E-19	-8.39	1
		protein	Pancreas	1.25E-12	-6.76	1	2.55E-15	-7.78	1
		protein	Pituitary	8.91E-13	-6.14	1	1.07E-11	-6.8	1
		protein	Small_Intestine_Terminal_Ileum	-	-	-	1.12E-21	-8.97	1
		protein	Spleen	3.89E-12	-8.19	1	6.88E-21	-9.22	1
		protein	Whole_Blood	6.03E-10	-7.19	1	1.44E-14	-8.03	1
4q21.1	CDKL2^ab^	protein	Stomach	3.92E-09	5.93	0.934	3.59E-09	6.05	0.957
5q12.1	NDUFAF2^ab^	protein	Brain_Cortex	1.11E-08	5.5	0.996	4.41E-09	5.65	0.998
		protein	Brain_Hippocampus	1.30E-08	5.46	0.997	5.27E-09	5.6	0.999
		protein	Brain_Nucleus_accumbens_basal_ganglia	6.97E-09	5.6	0.999	2.75E-09	5.75	0.999
		protein	Brain_Putamen_basal_ganglia	4.95E-09	5.68	0.999	1.92E-09	5.83	1
		protein	Brain_Substantia_nigra	2.15E-08	5.34	0.994	8.93E-09	5.48	0.997
		protein	Pancreas	-	-	-	1.90E-07	5.67	0.994
6p21.32	HLA-DQB1^b^	protein	Brain_Cortex	-	-	-	1.49E-06	-4.77	0.909
		protein	Stomach	-	-	-	1.49E-06	-4.77	0.911
11q25	IGSF9B^b^	protein	Brain_Cerebellar_Hemisphere	-	-	-	2.96E-07	-4.88	0.945
		protein	Brain_Hippocampus	-	-	-	2.38E-07	-4.92	0.954
		protein	Small_Intestine_Terminal_Ileum	-	-	-	4.18E-07	-4.82	0.927
12q24.31	CCDC62^ab^	protein	Brain_Cerebellar_Hemisphere	2.10E-08	5.76	0.999	1.11E-09	5.64	0.999
		protein	Brain_Cerebellum	-	-	-	4.98E-09	5.41	0.996
16p11.2	FBXL19^b^	protein	Brain_Substantia_nigra	-	-	-	1.17E-10	-6.18	0.966
17p12	TTC19^a^	protein	Whole_Blood	3.13E-07	-4.88	0.924	-	-	-
17p12	ADORA2B^b^	protein	Brain_Substantia_nigra	-	-	-	8.42E-08	5.04	0.969
		protein	Colon_Transverse	-	-	-	1.31E-07	5	0.964
17q21.31	ARHGAP27^ab^	protein	Brain_Caudate_basal_ganglia	1.10E-19	-9.26	1	1.75E-20	-9.53	0.985
17q21.32	CRHR1^a^	protein	Colon_Sigmoid	1.50E-21	-9.38	0.971	-	-	-
17q21.32	WNT3^ab^	protein	Colon_Transverse	8.50E-20	-8.51	0.962	1.48E-21	-8.8	0.985
		protein	Whole_Blood	5.73E-16	-6.89	1	-	-	-
3q27.1	MCCC1-AS1^ab^	lncRNA	Brain_Cerebellar_Hemisphere	2.36E-07	-4.1	0.937	-	-	-
		lncRNA	Brain_Cortex	5.80E-08	-4.71	0.963	6.13E-07	-4.12	0.915
		lncRNA	Brain_Frontal_Cortex_BA9	8.33E-08	-4.57	0.995	-	-	-
		lncRNA	Brain_Nucleus_accumbens_basal_ganglia	8.67E-08	-4.56	0.947	-	-	-
4q22.1	SNCA-AS1^b^	lncRNA	Brain_Cerebellar_Hemisphere	-	-	-	3.13E-06	-5.32	0.944
7p15.3	KLHL7-DT^b^	lncRNA	Brain_Cerebellum	-	-	-	2.75E-07	-4.79	0.919
17q21.31	MAPT-IT1^b^	lncRNA	Brain_Substantia_nigra	-	-	-	1.93E-22	-9.63	0.963
17q21.31	KANSL1-AS1^b^	lncRNA	Brain_Spinal_cord_cervical_c-1	-	-	-	5.00E-23	-9.68	0.981
		lncRNA	Liver	-	-	-	6.67E-21	-9.24	0.992
17q21.31	CR936218.1^a^	lncRNA	Stomach	3.87E-20	9.03	0.996	-	-	-
17q21.32	CR936218.2^a^	lncRNA	Liver	7.73E-21	8.21	1	-	-	-
	MAPT-AS1^a^	lncRNA	Brain_Amygdala	4.51E-08	9.38	0.944	-	-	-

^a^The posterior probabilities of FOCUS >0.9 only in PD GWAS.

^b^The posterior probabilities of FOCUS >0.9 only in PD MTAG.

^ab^the posterior probabilities of FOCUS >0.9 both in PD GWAS and PD MTAG.

### Comparison of PWAS, SMR, TWAS and FOCUS highlighted high confidence risk genes for PD

In summary, we identified 16 genes whose genetically regulated protein abundance levels were associated with PD risk by PWAS or SMR. Next, we explored the overlap of risk genes at the protein and RNA levels. Comparing PWAS, SMR, TWAS and FOCUS highlighted high confidence risk genes associated with PD. Through a succession of comparisons between the PWAS, SMR, TWAS and FOCUS results, 5 *(CD38*, *GPNMB*, *RAB29*, *TMEM175*, *TTC19*) of the 16 proteome-wide significant genes were supported by TWAS and FOCUS, suggesting that these genes hold the potential to become therapeutic targets for PD.

### Gene ontology (GO) and Kyoto Encyclopedia of Genes and Genomes (KEGG) pathway enrichment analyses

**Figure 3:**
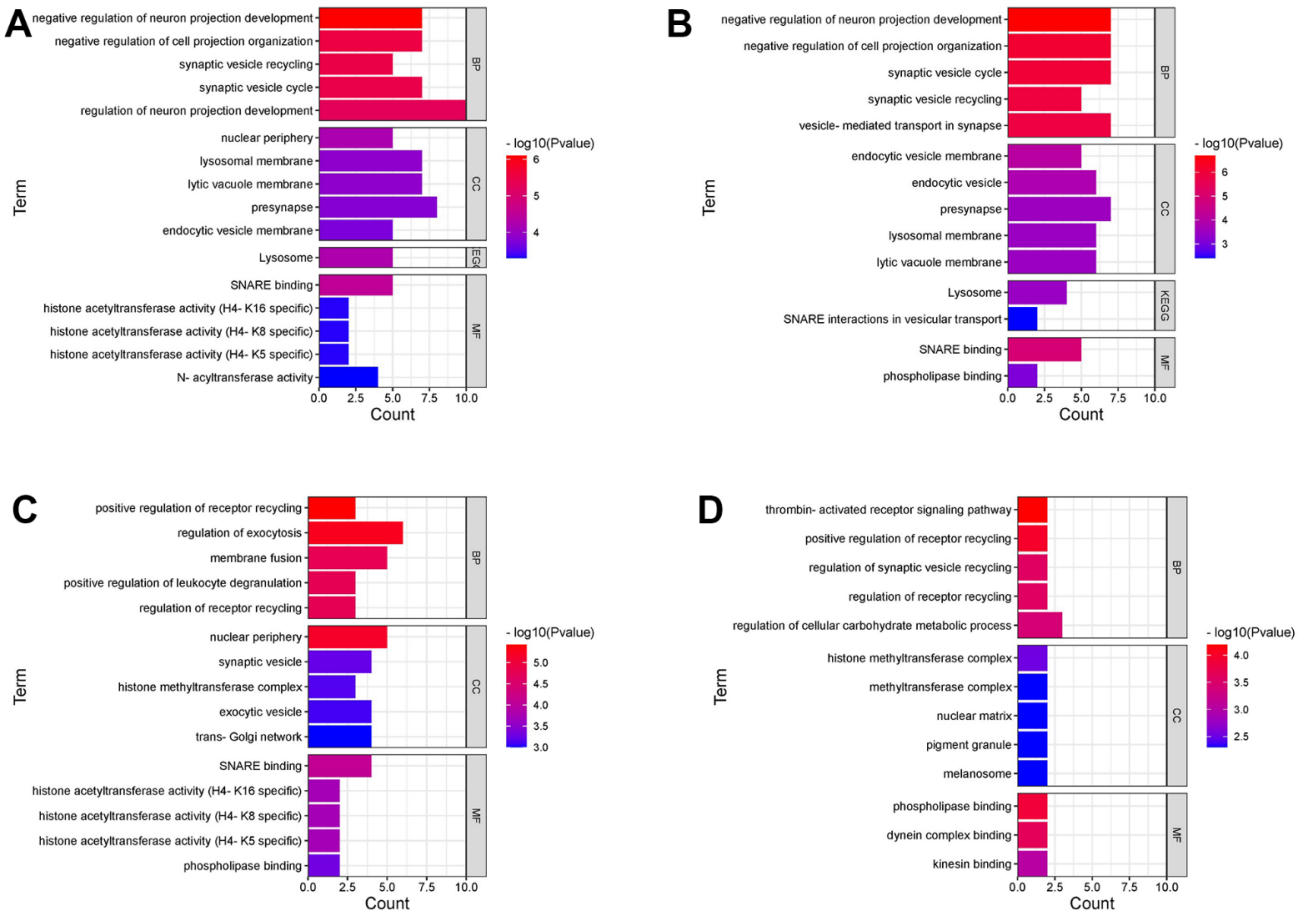
**GO and KEGG pathway enrichment analyses.** (**A**) GO and KEGG pathways are statistically significant of 95 genes associated with PD risk in TWAS. (**B**) GO and KEGG pathways are statistically significant of 79 genes associated with PD risk (TWAS) in fourteen tissues of central nervous systems. (**C**) GO pathways are statistically significant of 59 genes associated with PD risk (TWAS) in seven digestive system tissues. (**D**) GO pathways are statistically significant of 26 genes associated with PD risk (TWAS) in the whole blood. BP, biological processes; MF, molecular functions; CC, cellular components.

Seventy-nine genes related to PD risk identified by TWAS in the CNS were mainly enriched in the lysosomal pathway and SNARE interactions in the vesicular transport pathway by KEGG enrichment analysis (Supplementary Figure 5 and Supplementary Table 20). GO analysis explored the associated biological processes, molecular functions, and cellular components of the gene set. Detailed results of 22 tissues (Supplementary Figure 6 and Supplementary Table 21), digestive system (Supplementary Figure 7 and Supplementary Table 22), and whole blood (Supplementary Figure 8 and Supplementary Table 23) enrichment analysis are presented in the additional material. The associated genes in the CNS and their interactors mainly regulate neuron projection development, cell projection organization, and the synaptic vesicle cycle. However, the associated genes in the digestive system and their interactors mainly regulate receptor recycling and exocytosis. The associated genes in whole blood and their interactors mainly regulate the thrombin-activated receptor signaling pathway and receptor recycling (Figure 3).

## DISCUSSION

In this study, we performed PWAS and SMR analysis by integrating PD GWAS with proteome and pQTL data from human brain, plasma and CSF. We identified 16 genes whose genetically regulated protein abundance levels are associated with PD risk. Our study provides novel genetic insights into the pathogenic mechanisms of PD at the protein abundance level. Through TWAS and FOCUS analysis, we further identified 95 genes with transcriptional expression levels significantly associated with PD, and identified 27 potential causal genes by FOCUS analysis. Combining the results of PD and PD MTAG, we identified 26 new potential causal genes that were not previously reported in TWAS studies (Table 2).

Five genes including *CD38*, *GPNMB*, *RAB29*, *TMEM175*, and *TTC19* demonstrated significant associations with PD on both protein and mRNA level, suggesting that these genes are promising therapeutic targets with their gene-regulated expression and protein abundance together significantly correlated with PD. One of the five shared genes, *GPNMB* is widely expressed in whole brain regions and is important for brain aging. In a recent study, GPNMB was identified confer risk for PD through interaction with α-synuclein. Plasma GPNMB levels were also increased in PD patients, and PD patients with higher plasma GPNMB levels were more severe [35]. In our FOCUS analysis, for genomic locus 7:22508611-7:23469560, *GPNMB* was the only gene with a posterior probability of 0.931 in the 90%-credible gene set and further supported that *GPNMB* is a conformed risk gene for PD [36]. In FOCUS analysis, *RAB29* is indicated as a putative causal gene for PD in a total of 20 tissues, including the central nervous system and the digestive system. Multiple genetic studies have indicated that the small GTPase Rab29 is involved in the pathogenic mechanism of PD [37]. Through GO enrichment analysis of PD related genes in the CNS, it was discovered that *RAB29* is enriched in multiple pathways, including neuron projection development, cell projection organization, neuron differentiation, and more. However, by gene enrichment analysis of Parkinson’s disease-related genes in the digestive system, *RAB29* was mainly enriched in the receptor cycling pathway. By the FOCUS approach, *MMRN1* is likely a causal gene for PD risk in 16 tissues including the central nervous system and digestive system. Our study provides genetic support that the pathological process of PD involves all levels of the brain-gut axis.

Another important PWAS and TWAS shared gene is *TMEM175*. TMEM175 is a widely expressed lysosomal membrane protein that serves as a proton-activated, proton-selective channel, mediating lysosomal H+ efflux. TMEM175 deficiency is associated with impaired intracellular protein hydrolysis activity and the aggregation of α-synuclein [38, 39]. We also noticed enrichment of specific lysosome related pathways in the TWAS analysis in 79 identified genes based on CNS prediction models and 95 identified genes based on all tissues (Supplementary Figures 5, 6), supported the hypothesis that the genes associated with PD identified in this study are a subset of genes with similar biological functions and interactions, of which the lysosomal pathway play a crucial role in the pathogenesis of PD [40, 41].

Interestingly, we found that the prioritization of causal genes tends to vary in different tissues. We observed tissue-specific association directions among 10 genes, including *CRHR1*, *HSD3B7*, *LRRC37A*, *MAPT*, *MAPT*-*IT1*, *PLEKHM1*, *PRSS53*, *SNCA*, *STX4*, *VKORC1*, and *ZSWIM7*. For example, SNCA shows negative Z values in brain tissue, but positive Z values in the spleen. This may suggest that causal genes play different biological roles in the pathogenesis of PD across various tissues, warranting warrants further investigation.

Currently, the brain–spleen-gut axis is a crucial communication network for maintaining the body’s balance [42, 43]. Specifically, the spleen can influence the functionality of the brain through immune regulation. Studies have indicated that, in Alzheimer’s Disease mouse models, spleen macrophages directly clear peripheral Aβ [44]. PD affects all aspects of the brain–spleen-gut axis, interestingly, while the most common PD risk gene GBA has not been identified in previous TWAS studies using the brain model, we found that the expression level of GBA in spleen showed significant correlation with PD. Spleen is also affected in Gaucher’s disease with accumulation of glucocerebroside due to loss of function mutations of GBA. These results may suggest that the mechanism of GBA deficiency leading to PD may occur in not only central nervous system but also peripheral organs such as spleen, and more experimental evidence needs to clarify this hypothesis.

In the PD MTAG analysis, we identified more risk genes supporting the validity that MTAG boosts the statistical power to detect the novel disease associated genes for PD. Combined with the results of PD and PD MTAG, 26 novel PD associated genes have been identified (Table 2), including eight long noncoding RNA (lncRNA), *MCCC1*-*AS1*, *SNCA-AS1, KLHL7*-*DT*, *MAPT*-*IT1*, *KANSL1*-*AS1*, *MAPT-AS1*, *CR936218*.*1*, and *CR936218*.*2* and 18 protein-coding genes. Recently, an increasing number of studies have reported the involvement of lncRNA in the pathogenesis of PD. Circulating lncRNA levels may serve as biomarkers for PD [45]. LncRNAs play an important role in the pathogenesis of PD, affecting dopaminergic neuron survival, autophagy, mitochondrial function, and inflammatory responses in different ways [46]. In the other 18 encoding genes, some encoded proteins are associated with mitochondrial. TTC19 serves as a subunit of mitochondrial respiratory chain Complex III, and it is an essential component for the assembly and activation of complex III [47]. Patients with loss of function mutations in *TTC19* develop progressive encephalopathy associated with a deficiency in complex III. In addition, NDUFAF2 was identified as a potential complex I assembly factor [48]. The loss of function of *NDUFAF2* could cause mitochondrial encephalopathy, and there are some cases of complex I-associated mutations resulting in Parkinsonism or substantia nigra pars compacta-selective neurodegeneration regulation. More evidence from cellular and animal models are further needed to clarify the relationship between new identified genes and PD.

Some potential limitations still need to be acknowledged when interpreting our findings. Firstly, only part of the PWAS and SMR genes were supported by TWAS and observed that the number of risk genes identified by PWAS and SMR are less than TWAS, that could partly be explained by the uncorrelated changes in mRNA and protein expression levels [28], and limited individual samples used for protein weights generation. Secondly, this study employed genetic and statistical analysis methods for the identification of risk genes. Further experimental work is required to better elucidate whether the identified genes play a causal role in the pathogenesis of PD. Further verification is required for genes that have shown inconsistent association directions in this study and in previous studies. Thirdly, due to the utilization of European linkage disequilibrium structures in the Fusion software run under default settings, the PWAS, SMR, TWAS and FOCUS analyses in this study are limited to individuals of European ancestry. We need to conduct more studies with different ancestries to verify our results. Finally, we listed the P-values of significant genes obtained by TWAS method using PD MTAG or PD GWAS data sets (Supplementary Table 24). We found that risk genes identified by TWAS tended to have smaller p-values using PDMTAG data, but there were few contrary results. Despite the limited size of the MTAG data, we have endeavored to highlight the unique contributions of our study and the potential for PWAS to complement and enrich the findings of TWAS. We sincerely hope that our study, despite its apparent limitations, will be seen as a valuable addition to the ongoing efforts to understand Parkinson’s disease.

In summary, we identified 16 genes whose genetically regulated protein abundance levels in the human brain, CSF or plasma are associated with PD risk. We undertook a large-scale analysis of PD and correlated traits, through TWAS and FOCUS studies, we discovered 26 causal genes related to PD that had not been reported in previous TWAS studies. This study reveals the pathogenesis of PD from genetics, transcriptome, proteomics and other levels, and lays the foundation for further research on related molecular mechanisms and intervention targets.

## MATERIALS AND METHODS

### Datasets used in this study

### GWAS dataset

This study was analyzed and investigated using three GWAS data, which included information on the rsID, base pair position, and P-value of SNPs, and all case-control individuals were from European populations.

The PD GWAS dataset used in this study was sourced from the International Parkinson’s Disease Genomics Consortium, which conducted a large-scale meta-analysis by combining 14 GWAS datasets, including 33,674 PD cases and 449,056 controls [10]. PD GWAS data was obtained from public websites (https://gwas.mrcieu.ac.uk/datasets/ieu-b-7/). The LBD GWAS dataset was from 2,591 individuals diagnosed with LBD and 4,027 healthy controls with participants recruited from 44 institutions and diagnosed with Lewy body dementia according to established consensus criteria (https://www.ebi.ac.uk/gwas/publications/33589841) [49]. The GWAS of iRBD data comprised 1,061 cases and 8,386 controls [50]. This iRBD cohort includes a large number of French, French-Canadian, Italian, and British origin, as well as other smaller cohorts from different European populations (https://www.tinyurl.com/iRBDsumStats).

### pQTL data

We used the previous studies generated human brain proteomes in this study (Supplementary Table 25). Briefly, a study performed a proteome analysis using brain tissues from the dorsolateral prefrontal cortex (dlPFC) of 376 human subjects from Religious Order Study and Rush Memory and Aging Project (ROSMAP dataset) [51]. 1475 proteins were used for PWAS (https://doi.org/10.7303/syn23627957). In addition, this study further validated the result by using the brain proteome from the Banner dataset [52]. After quality control, brain proteomes of 152 participants were available for proteome analysis. Following the analysis, 1139 proteins showed significant associations with genetic variations (https://doi.org/10.7303/syn23627957) [53]. Another study analyzed 4,657 plasma proteins data from 7,213 European American participants in the ARIC study. In our research, we used the consequences of 2,004 proteins of European American (EA) ancestry for PWAS. A recent study measured the abundance of 1,305 proteins in CSF (n=971), plasma (n=636), and brain (n=458) samples [54]. We conducted rigorous quality control on the proteomic data. After QC, 8 CSF proteins and 16 plasma proteins showed significant cis associations with genetic variation (weights).

### GTEx eQTL data

The most complete eQTL database to date is GTEx, which performs simultaneous transcriptome sequencing and genotyping of multiple tissues from normal humans with the aim of establishing associations between genotype and gene expression levels. GTEx version 8 (V8) covers 17,382 samples sequenced from 54 human tissues from 948 donors. GTEx uses both the gene expression data and the genotype data for eQTL analysis to determine the relationship between each gene and its expression level, and stores the results on its website (https://www.gtexportal.org/home/). Zhou et al. used joint-tissue imputation (JTI) method to integrate GTEx eQTL data [32]. Previous training of predictive expression models (PrediXcan, UTMOST), underutilized the extensive biological similarity between tissues of GTEx data. JTI expression prediction model is a model that uses data from multiple tissue samples to predict the expression of a single tissue sample. JTI combines the information from multiple similar tissues that can improve the accuracy and reliability of TWAS analysis, and thereby better revealing the association between genes and phenotypes and providing new ideas for the prevention and treatment of related diseases. We downloaded the JTI gene expression prediction model data, which includes eQTL summary statistics and SNP-SNP covariance matrix at (https://zenodo.org/record/3842289).

### Methods and software

### Multi-trait analysis of GWAS (MTAG)

MTAG conducts a joint analysis of multiple traits by integrating the genetic correlation structure of several similar traits into a single ‘meta-analysis,’ thereby improving the efficiency of discovering associated genes. The MTAG method allows for the pooling of data from multiple GWAS studies without increasing additional computational costs, leading to an increased sample size. This, in turn, enhances the ability to detect genetic variations and reduces the risk of false positives or negatives arising from genetic correlations [22]. We incorporated 2 GWAS (LBD, iRBD) of traits correlated with PD in MTAG to identify additional SNPs associated with PD risk (https://github.com/JonJala/mtag).

### Proteome-wide association studies (PWAS)

We used the Fusion package to perform PWAS (http://nilanjanchatterjeelab.org/pwas) [55]. Briefly, we obtained GWAS data and protein expression prediction models for human brain (ROSMAP/Banner), plasma and CSF [53]. Utilizing the ROSMAP/Banner/plasma/CSF protein abundance-weighted prediction model, we predicted the protein expression levels of each gene in different tissues. Finally, we performed association analysis using the FUSION software on protein abundance and disease phenotype data to determine the associations between predicted protein abundance and PD. We corrected the results of PWAS using the Bonferroni method (0.05/the number of genes included in PWAS).

### Summary data-based mendelian randomization (SMR)

This study further employed the Summary data-based Mendelian Randomization method to validate and complement the results obtained from PWAS. The analysis was conducted using the SMR analysis program available on the SMR website (downloaded from https://yanglab.westlake.edu.cn/software/smr) [31]. We utilized the SMR method to perform linear regression on large-scale GWAS and pQTL data, conducting causal relationship validation in a large sample size, which contributes to increased statistical power. We employed the HEIDI method, a tool for testing heterogeneity in result association statistics. We used an unadjusted P ≤ 0.01 to indicate that the presence of heterogeneity affected the SMR results.

### Gene-based association analysis using S-PrediXcan

In this study, TWAS was performed using S-PrediXcan software [56, 57] (https://github.com/hakyimlab/MetaXcan). Firstly, GWAS data and JTI gene expression prediction model were obtained. Secondly, the effect sizes of SNPs in GWAS were converted to the effect sizes of gene expression. Finally, the predicted gene expression was analyzed in correlation with the phenotypes, and the correlation results were obtained between each gene and the phenotypes.

The JTI expression prediction model is a model that utilizes data from multiple tissue samples to predict the expression of a single tissue sample. JTI combines information from multiple similar tissues can improve the accuracy and reliability of TWAS analysis, thereby better revealing the association between genes and phenotypes and providing new ideas for the prevention and treatment of related diseases. We used 22 tissue-specific expression models (PredictDB; http://predictdb.org) to represent the nervous system (amygdala, anterior cingulate cortex BA24, caudate basal ganglia, cerebellar hemisphere, cerebellum, cortex, frontal cortex BA9, hippocampus, hypothalamus, nucleus accumbens basal ganglia, putamen basal ganglia, spinal cord cervical c-1, substantia nigra, pituitary), digestive system (sigmoid, transverse, liver, pancreas, small intestine terminal ileum, spleen, stomach) and the whole blood. We corrected the results of TWAS using the Bonferroni method (0.05/the number of genes included in TWAS).

### Fine-mapping of TWAS associations

Fine-mapping Of Causal Gene Sets, FOCUS, is based on two assumptions [33, 34]:

1. The credible set assumption: variation in gene expression is usually due to a set of jointly acting genetic variants that are concentrated within some specific region (i.e., the credible set).
2. Causal hypothesis: only a small fraction of the genetic variants within the plausible set are actually responsible for the change in expression (i.e., causal variants), while the rest of the genetic variants may simply be closely associated with the causal variants.

Due to the presence of linkage disequilibrium among SNPs used to construct expression weights, transcriptome imputation methods (such as S-PrediXcan) may be prone to false-positive gene-trait associations. To address co-regulation in TWAS, FOCUS was used to identify those genetic variants that may have a functional impact on gene expression by fine-tuning these regions (https://github.com/bogdanlab/focus/).

By integrating GWAS data, gene expression prediction weight data (JTI) created based on eQTL data from multiple tissues, and linkage disequilibrium data for all SNPs in regions susceptible to genetic mutations, we predicted causal genes contained in 90% confidence intervals, computed the Posterior Inclusion Probability (PIP) for each gene in the region of a genetic mutation associated with disease. The PIP is a probability value used to measure the degree of association between each SNP and the phenotype. The PIP value ranges from 0 to 1, with higher values indicating a stronger association between the SNP and the phenotype. The significance thresholds in the present study were set to genes within the 90% confidence interval and a posteriori probability values ≥ 0.9 [33, 34].

### GO and KEGG pathway enrichment analyses

We conducted GO and KEGG pathway enrichment analyses using the R package ‘clusterProfiler’ to explore the potential functional pathways of susceptibility genes (https://yulab-smu.top/biomedical-knowledge-mining-book/). GO is a standardized system for describing the functions of genes and their encoded products, which usually involves three levels: molecular function (MF), cellular component (CC), and biological process (BP) [58]. Through GO enrichment analysis, we can have a preliminary understanding of the biological functions, pathways or cellular localizations in which genes associated with Parkinson’s disease are enriched. The R package “clusterProfiler” (https://yulab-smu.top/biomedical-knowledge-mining-book/) was utilized to perform GO and KEGG pathway enrichment analysis to explore the potential functions pathways of susceptibility genes.

### Data sharing

PD GWAS summary statistics by Nalls et al. can be downloaded under https://gwas.mrcieu.ac.uk/datasets/ieu-b-7/. LBD GWAS summary statistics by Chia et al. can be downloaded under https://www.ebi.ac.uk/gwas/home [49]. iRBD GWAS summary statistics by Lynne Krohn et al. can be downloaded under www.tinyurl.com/iRBDsumStats [50]. The ROSMAP dataset and Banner dataset (weights and pQTL) are available from https://doi.org/10.7303/syn23627957 by Wingo et al. [53]. The JTI prediction models GTEx models can be downloaded from Zenodo (https://doi.org/10.5281/zenodo.3842289). The Plasma dataset is available from http://nilanjanchatterjeelab.org/pwas. For the three tissues (CSF, brain, plasma) in the Knight ADRC dataset, both summary statistics and individual-level data are available from https://www.niagads.org/datasets/ng00102. The code can be found on https://github.com/Anonymousxyee/Joint-Analysis-of-PWAS--TWAS--and-Multi-Trait-Analysis-to-Identify-Novel-PD-Risk-Gene.

## Supplementary Material

Supplementary Figures

Supplementary Tables 1 and 2

Supplementary Table 3

Supplementary Table 4

Supplementary Table 5

Supplementary Table 6 and 7

Supplementary Table 8

Supplementary Table 9

Supplementary Table 10

Supplementary Table 11

Supplementary Table 12

Supplementary Table 13

Supplementary Table 14

Supplementary Table 15

Supplementary Table 16

Supplementary Table 17

Supplementary Table 18

Supplementary Table 19

Supplementary Table 20

Supplementary Table 21

Supplementary Table 22

Supplementary Table 23

Supplementary Table 24

Supplementary Table 25
