# Supplementary material for: Joint analysis of proteome, transcriptome, and multi-trait analysis to identify novel Parkinson’s disease risk genes
Source: Aging (Albany NY). 2024 Jan 17;16(2):1555–80. doi: 10.18632/aging.205444 (PMC10866412; doi:10.18632/aging.205444)
Supplement: Supplementary Figures [file aging-16-205444-s001.pdf]

SUPPLEMENTARY FIGURES

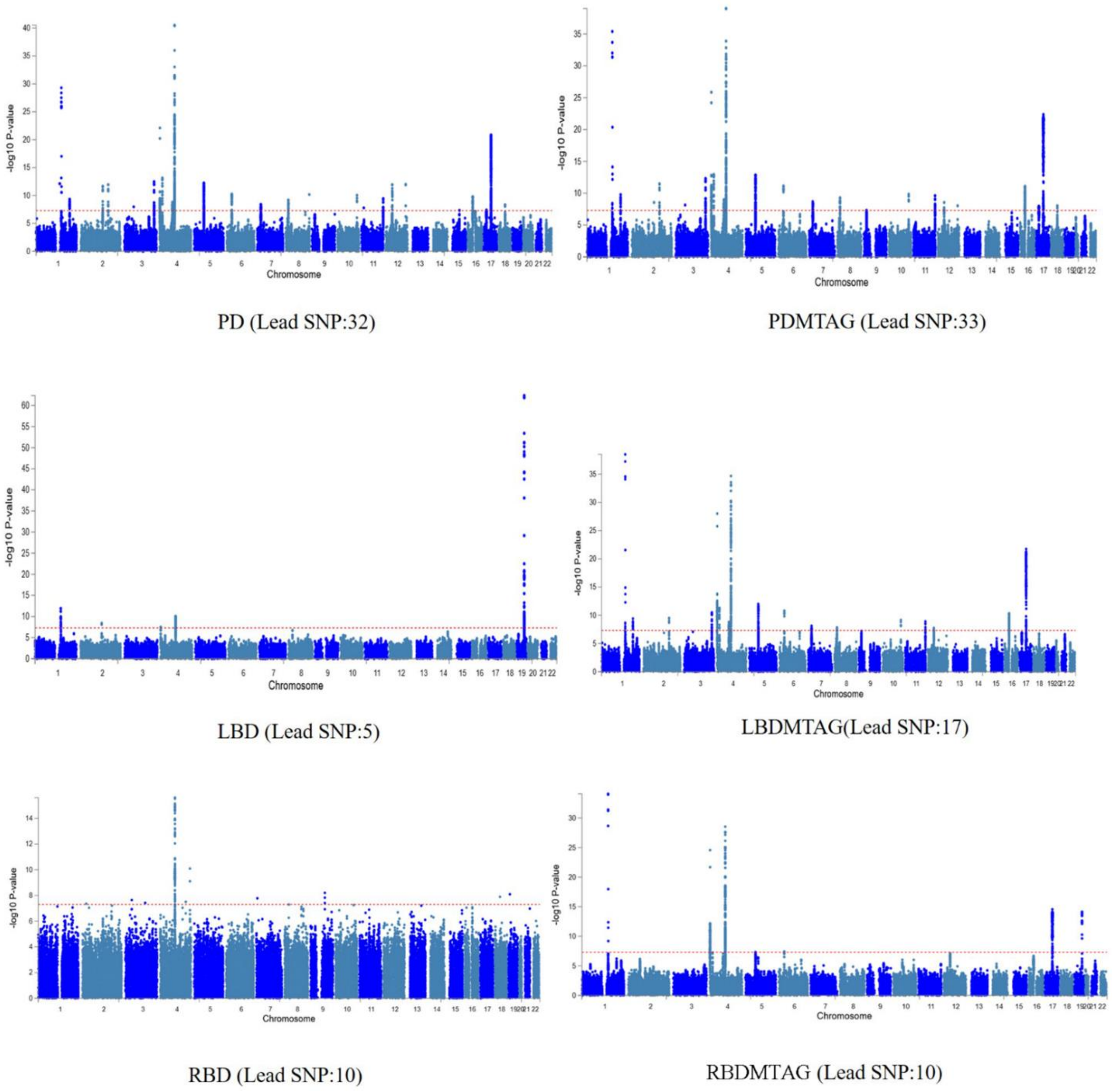

Supplementary Figure 1. Manhattan plots for individual GWAS and MTAG.

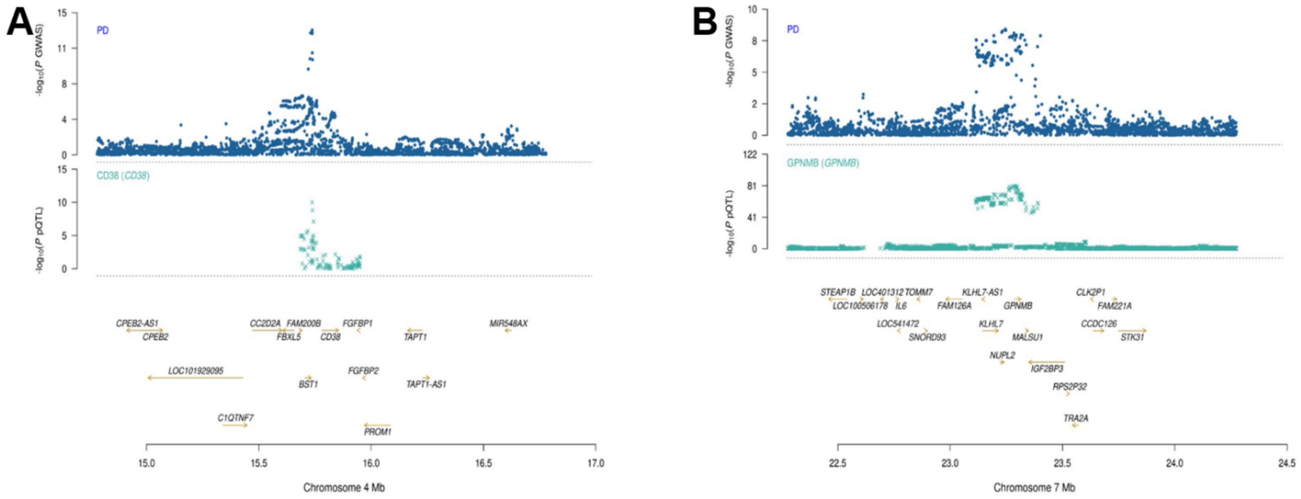

Supplementary Figure 2. Prioritizing genes at a GWAS locus using SMR analysis. (**A**) Shown are results at the CD38 locus for PD. (**B**) Shown are results at the GPNMB locus for PD. Top plot, brown dots represent the P-values for SNPs from the latest GWAS meta-analysis for PD. The top and bottom plots include all the SNPs available in the region in the GWAS and pQTL summary data, respectively, rather than only the SNPs common to both data sets. Top plot is the gene that passed the SMR and HEIDI tests.

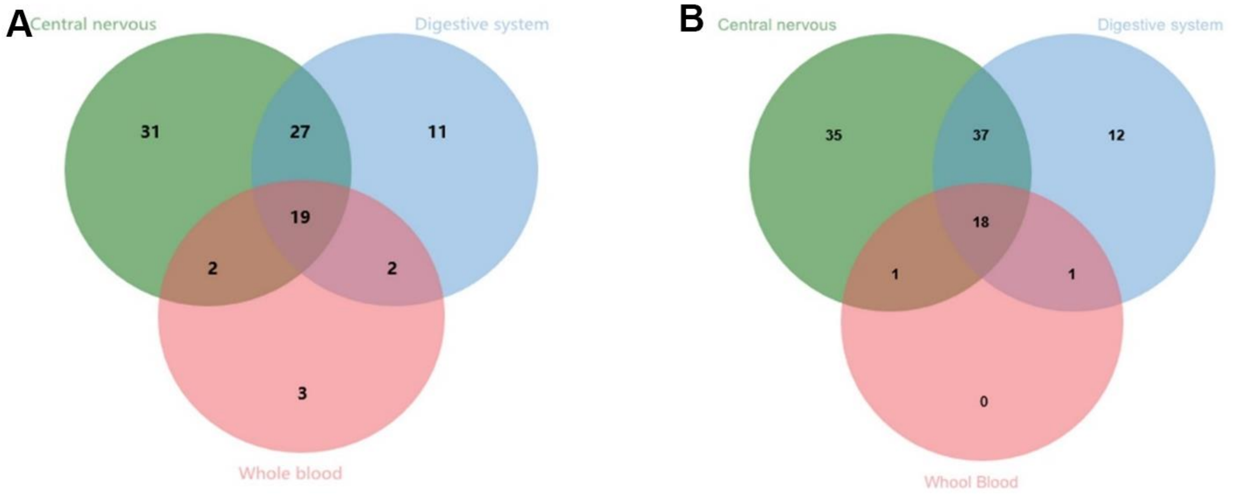

Supplementary Figure 3. Venn plots of the PD significant genes. (**A**, **B**) Venn plots of PD risk genes identified in central nervous system, digestive system and blood system by TWAS (JTI models). (**A**) was PD GWAS. (**B**) was in PD MTAG. Each round represents a study. Numbers in each overlapped area mean numbers of differently expression genes in each area. The intersection in the middle represents genes which are significantly differentially expressed in three studies consistently.

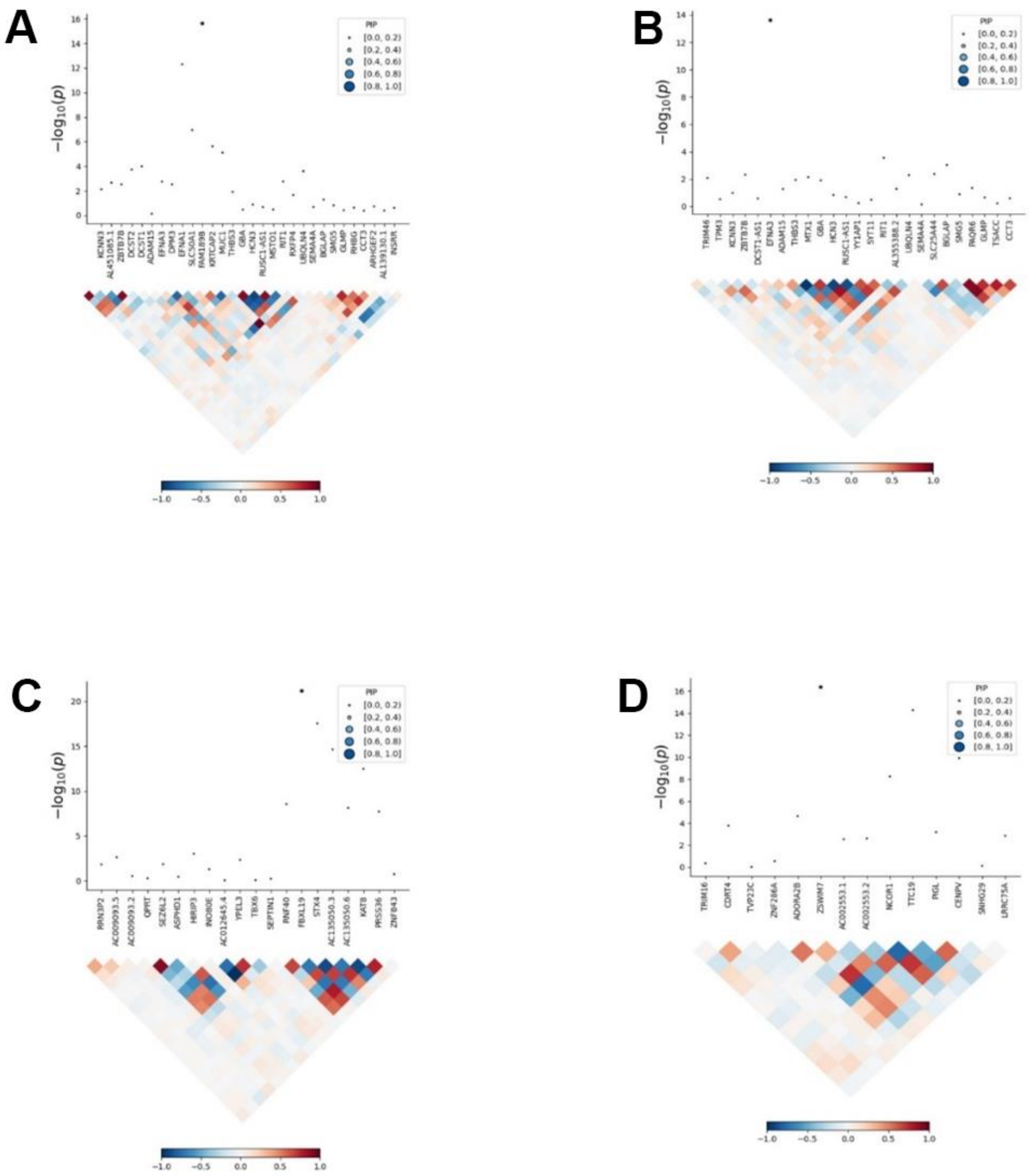

Supplementary Figure 4. FOCUS plot for each gene in one region. (**A**) The plot contains the predicted expression correlation, TWAS summary statistics and PIP for each gene in the genomic locus 3:181511166-3:183769683 in the brain cerebellum. (**B**) The plot contains the predicted expression correlation, TWAS summary statistics and PIP for each gene in the genomic locus 3:181511166-3:183769683 in the stomach. (**C**) The plot contains the predicted expression correlation, TWAS summary statistics and PIP for each gene in the genomic locus16:29036613-16:31382470 in the brain substantia nigra. (**D**) The plot contains the predicted expression correlation, TWAS summary statistics and PIP for each gene in the genomic locus 17:15020965-17:16411522 in the whole blood.

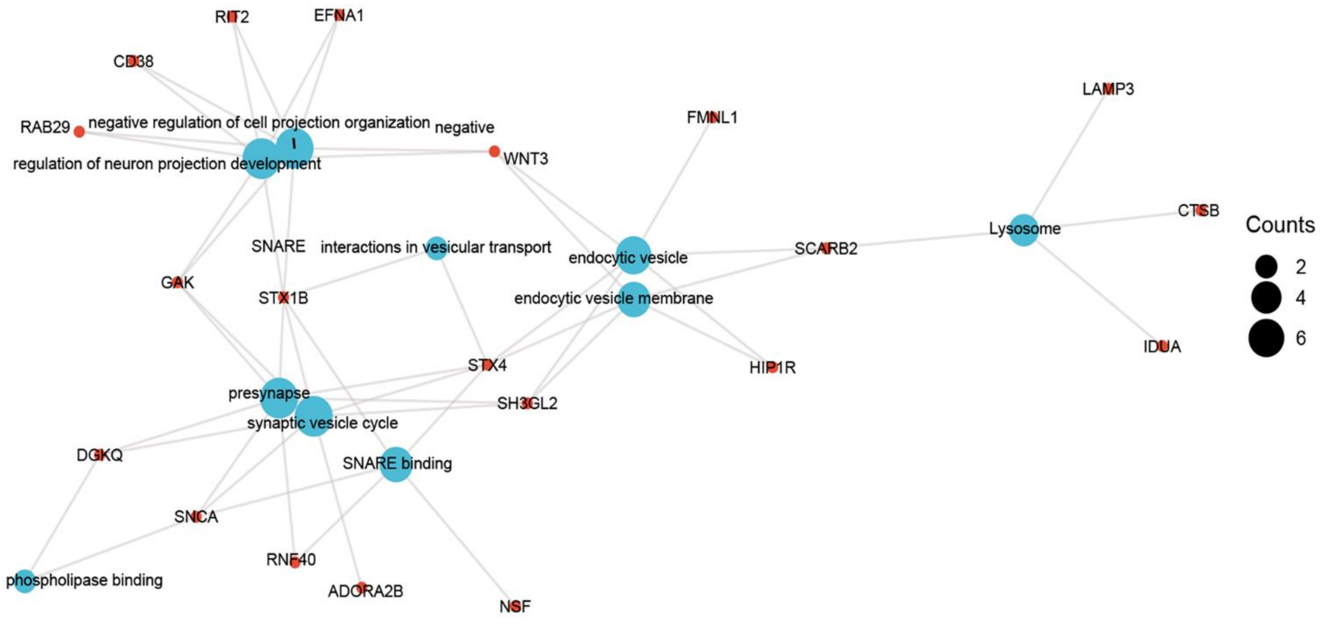

Supplementary Figure 5. Interaction network analyses of the 79 genes associated with PD risk in TWAS using fourteen tissues of central nervous systems. Each blue node represents a functional pathway or cell type and the red node represents a gene. The connections represent the interaction between a functional pathways or cell type with a gene.

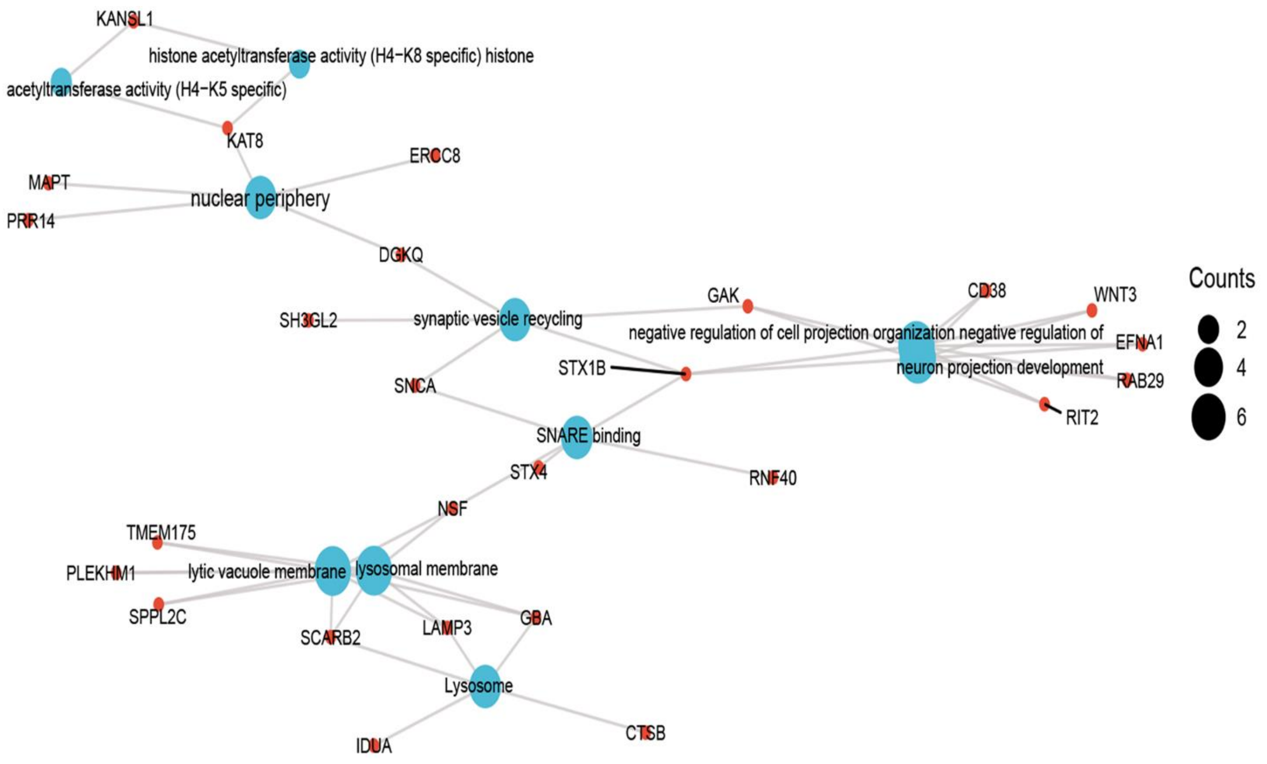

Supplementary Figure 6. Pathway interaction network diagram of 95 genes found by TWAS in 22 tissues.

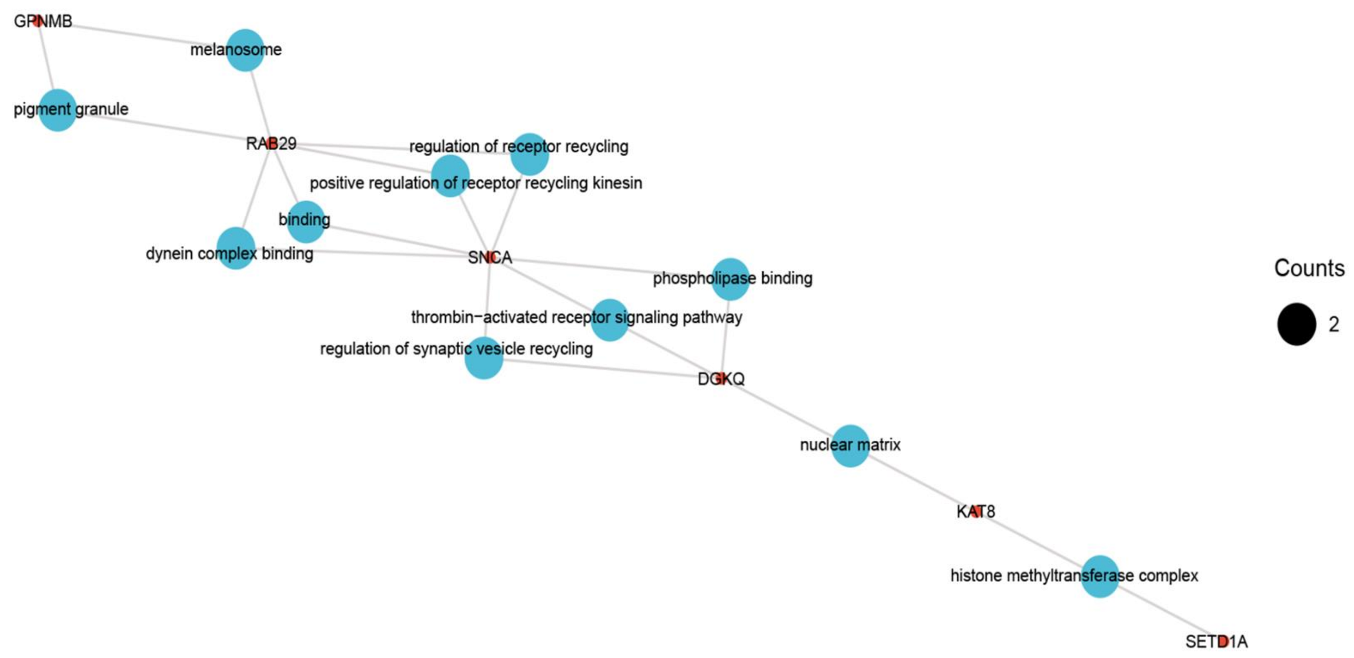

Supplementary Figure 7. Pathway interaction network diagram of 26 genes found by TWAS in the whole blood.

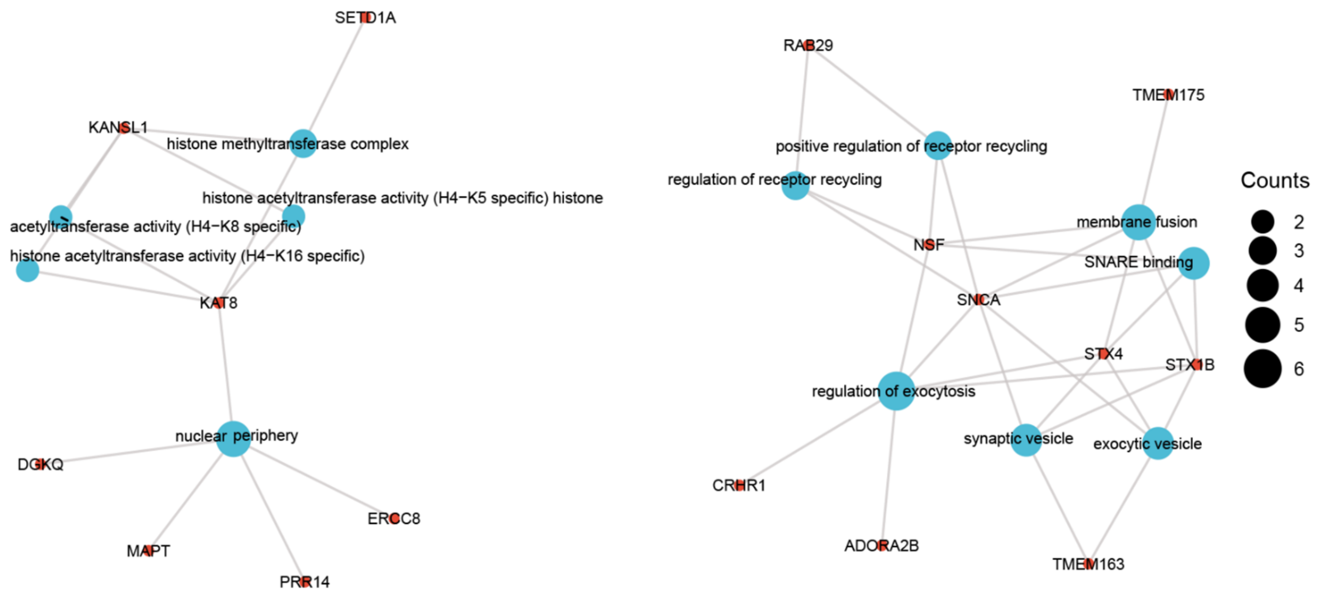

Supplementary Figure 8. Pathway interaction network diagram of 59 genes found by TWAS in 7 tissues in digestive system.
