# Supplementary material for: Joint analysis of proteome, transcriptome, and multi-trait analysis to identify novel Parkinson’s disease risk genes
Source: Aging (Albany NY). 2024 Jan 17;16(2):1555–80. doi: 10.18632/aging.205444 (PMC10866412; doi:10.18632/aging.205444)
Supplement: Supplementary Tables 1 and 2 [file aging-16-205444-s002.pdf]

SUPPLEMENTARY TABLES

Supplementary Table 1. Comparison of three previous TWAS studies.

	GWAS datasets	Transcriptomics panels	Methods	Significance genes
PMID: **30824768**	9581 cases and33245 controls	DLPFC and monocyte expression data	TWAS(fusion)	TMED5,RAB7L1,RAB4A,TMEM163,NMD3,CD38,MMRN1,GPNMB,DFNA5,CTSB,PDLIM2,FRA10AC1,GPR180,ATG14 ,NUDT14 ,HSD3B7,MED13,MEI1 ,GBP7, PTPN22, CLASP2, GNB4, NCK1, BST1, IDUA, SNCA, TMEM175,TRIML1, CAMLG, GGCT, GPNMB, PILRB, CTSB, MTMR9, CC2D2B, MAPK8IP1, LRRK2, GPR65, METTL3, NUDT14 , PSMC6, MAPK3, VKORC1, TNFSF13, CD33, SPPL2B, MAP1LC3A ,
PMID: **33523105**	26035 cases and 403190 controls	Braineac eQTL Data(10 brain regions) GTEx eQTL Data(13 brain regions)	TWAS(fusion)	WDR6,CD38,GPNMB,RAB29, and TMEM163
PMID: **34504106**	15,056 PD cases, 18,618 UK Biobank proxy-cases, and 449,056 controls	GTEx V8(13 brain regions)	TWAS(fusion)	LRRC37A2,LRRC37A,MMRN1,ARL17A,PLEKHM1,FMNL1,CD38, RNF40,SPPL2C,VKORC1,CCDC189,GPNMB,NUPL2,MAP3K14,ZSWIM7,GAK,CENPV,CPLX1,
This study	33,674 PD cases and 449,056 controls.	ROSMAP/Banner/plasma/CSF	PWAS/SMR/TWAS	16 genes protein abundance levels associated with PD risk. 95 gene expression levels associated with PD risk

Supplementary Table 2. Statistical summary of GWAS and MTAG results.

	PD	PDMTAG	LBD	LBDMTAG	RBD	RBDMTAG
Lead SNPs	32	33	5	17	10	10
Ind. Sig. SNPs	91	90	10	25	16	39
X^2	1.15	1.157	1.009	1.104	1.007	1.037
Sample size	482730	504827	7372	89741	9447	46816
