# Supplementary material for: Joint analysis of proteome, transcriptome, and multi-trait analysis to identify novel Parkinson’s disease risk genes
Source: Aging (Albany NY). 2024 Jan 17;16(2):1555–80. doi: 10.18632/aging.205444 (PMC10866412; doi:10.18632/aging.205444)
Supplement: Supplementary Table 6 and 7 [file aging-16-205444-s006.pdf]

Supplementary Table 6. The PWAS of PD integrating the CSF with the PD GWAS.

ID	CHR	P0	P1	BEST.GWAS.ID	BEST.GWAS. Z	EQTL.ID	EQTL.GWAS. Z	TWAS.Z	TWAS.P
PRDX6	1	173000000	174000000	rs2096147	-4.3	rs1234315	0.739	-0.739	0.46
CPE	4	166000000	167000000	NA	NA	NA	NA	NA	NA
PRSS1	7	142000000	143000000	rs6979469	-2.61	rs3752404	0.639	-0.639	0.523
EPHB6	7	142000000	143000000	rs6979469	-2.74	rs2272255	-0.443	0.443	0.658
PDCD1LG2	9	5010545	6010545	rs10815236	-3.61	rs10975371	0.714	0.714	0.475
CD274	9	4950503	5950503	NA	NA	NA	NA	NA	NA

Supplementary Table 7. The PWAS of PD integrating the plasma (Yang et al.) with the PD GWAS.

ID	CHR	P0	P1	BEST.GWAS.ID	BEST.GWAS. Z	EQTL.ID	TWAS.Z	TWAS.P
TNFSF18	1	172509100	173509100	NA	NA	NA	NA	NA
TNFSF4	1	172652873	173652873	rs2096147	-4.3	rs10158707	-1.112	0.266
PRDX6	1	172946405	173946405	rs2096147	-4.3	rs10158707	0.208	0.835
CD207	2	70557347	71557347	rs3771444	-3.67	rs3821261	-0.161	0.872
GZMA	5	53898476	54898476	rs1423249	2.81	rs7721054	1.19	0.234
TNFSF15	9	117046915	118046915	rs2104771	-3.31	rs2636897	0.878	0.38
CNTN1	12	40586244	41586244	rs1491932	-4.79	rs12370996	1.43	0.153
LDLR	19	10700038	11700038	rs3745682	-2.84	rs4804149	-0.811	0.417
ICAM3	19	9944452	10944452	rs3087689	-3.12	rs5030390	1.134	0.257
CD209	19	7304879	8304879	rs11672993	3.46	rs2303112	0.221	0.825
RETN	19	7233930	8233930	rs11672993	3.46	rs2303112	0.341	0.733
FCER2	19	7253644	8253644	rs11672993	3.46	rs2303112	0.455	0.649
CDC37	19	10001810	11001810	rs3087689	-3.12	rs5030390	0.659	0.51
ICAM1	19	9881511	10881511	rs3087689	-3.12	rs5030390	1.069	0.285
