# Supplementary material for: Joint analysis of proteome, transcriptome, and multi-trait analysis to identify novel Parkinson’s disease risk genes
Source: Aging (Albany NY). 2024 Jan 17;16(2):1555–80. doi: 10.18632/aging.205444 (PMC10866412; doi:10.18632/aging.205444)
Supplement: Supplementary Table 9 [file aging-16-205444-s008.pdf]

Supplementary Table 9. Internal performance of gene expression genetic prediction models of fourteen tissues of central nervous systems, seven tissues of the digestive system and the whole blood tissue.

Model	Prediction performance (R^2)	All	Protein	lncRNAs	miRNA
JTI -Brain_Amygdala	≥0.01	6,542	4,803	1,739	0
	≥0.04	5,734	4,176	1,558	0
	≥0.09	3,300	2,332	968	0
	≥0.16	1,770	1,203	567	0
JTI-Brain_Anterior_cingulate_cortex_BA24	≥0.01	7,735	5,740	1,995	0
	≥0.04	6,552	4,814	1,738	0
	≥0.09	3,910	2,778	1,132	0
	≥0.16	2,191	1,509	682	0
JTI-Brain_Caudate_basal_ganglia	≥0.01	10,121	7,626	2,494	1
	≥0.04	7,777	5,807	1,970	0
	≥0.09	4,625	3,418	1,207	0
	≥0.16	2,598	1,867	731	0
JTI-Brain_Cerebellar_Hemisphere	≥0.01	10,827	8,326	2,499	2
	≥0.04	8,903	6,858	2,043	2
	≥0.09	5,744	4,393	1,350	1
	≥0.16	3,571	2,724	846	1
JTI-Brain_Cerebellum	≥0.01	11,813	9,077	2,732	4
	≥0.04	9,279	7,174	2,103	2
	≥0.09	6,005	4,646	1,358	1
	≥0.16	3,697	2,835	861	1
JTI-Brain_Cortex	≥0.01	10,508	7,942	2,566	0
	≥0.04	7,987	6,000	1,987	0
	≥0.09	4,850	3,592	1,258	0
	≥0.16	2,829	2,053	776	0
JTI-Brain_Frontal_Cortex_BA9	≥0.01	9,288	6,965	2,323	0
	≥0.04	7,358	5,476	1,882	0
	≥0.09	4,468	3,256	1,212	0
	≥0.16	2,547	1,809	738	0
JTI-Brain_Hippocampus	≥0.01	8,159	6,074	2,084	1
	≥0.04	6,497	4,804	1,692	1
	≥0.09	3,648	2,650	997	1
	≥0.16	1,943	1,361	582	0
JTI-Brain_Hypothalamus	≥0.01	8,651	6,375	2,274	2
	≥0.04	6,639	4,868	1,770	1
	≥0.09	3,659	2,601	1,057	1
	≥0.16	1,952	1,344	608	0
JTI-Brain_Nucleus_accumbens_basal_ganglia	≥0.01	10,054	7,510	2,544	0
	≥0.04	7,488	5,536	1,952	0
	≥0.09	4,332	3,138	1,194	0
	≥0.16	2,423	1,715	708	0
JTI-Brain_Putamen_basal_ganglia	≥0.01	9,089	6,956	2,133	0
	≥0.04	7,339	5,599	1,740	0
	≥0.09	4,404	3,311	1,093	0
	≥0.16	2,462	1,783	679	0
JTI-Brain_Spinal_cord_cervical_c-1	≥0.01	7,868	5,881	1,987	0
	≥0.04	6,996	5,188	1,808	0

	≥0.09	3,959	2,861	1,098	0
	≥0.16	2,196	1,561	635	0
JTI-Brain_Substantia_nigra	≥0.01	6,616	4,880	1,735	1
	≥0.04	6,163	4,535	1,628	0
	≥0.09	3,534	2,500	1,034	0
	≥0.16	1,860	1,266	594	0
JTI-Colon_Sigmoid	≥0.01	12,370	9,572	2,797	1
	≥0.04	7,816	6,008	1,807	1
	≥0.09	4,505	3,403	1,101	1
	≥0.16	2,526	1,867	659	0
JTI-Colon_Transverse	≥0.01	12,827	9,791	3,032	4
	≥0.04	7,630	5,806	1,822	2
	≥0.09	4,265	3,225	1,038	2
	≥0.16	2,303	1,712	589	2
JTI-Liver	≥0.01	8,288	6,467	1,819	2
	≥0.04	5,623	4,329	1,292	2
	≥0.09	3,000	2,271	728	1
	≥0.16	1,552	1,148	403	1
JTI-Pancreas	≥0.01	10,964	8,800	2,162	2
	≥0.04	7,155	5,761	1,393	1
	≥0.09	4,181	3,339	841	1
	≥0.16	2,421	1,936	485	0
JTI-Pituitary	≥0.01	11,226	8,220	3,002	4
	≥0.04	7,685	5,538	2,146	1
	≥0.09	4,382	3,100	1,281	1
	≥0.16	2,496	1,758	737	1
JTI-Pituitary	≥0.01	11,603	8,819	2,782	2
	≥0.04	8,565	6,514	2,050	1
	≥0.09	5,116	3,865	1,250	1
	≥0.16	2,979	2,232	746	1
JTI-Stomach	≥0.01	11,366	8,831	2,534	1
	≥0.04	6,622	5,111	1,510	1
	≥0.09	3,538	2,718	819	1
	≥0.16	1,899	1,436	463	0
JTI-Whole_Blood	≥0.01	10,350	8,595	1,753	2
	≥0.04	6,177	5,155	1,022	0
	≥0.09	3,666	3,043	623	0
	≥0.16	2,110	1,765	345	0
JTI-Nerve_Tibial	≥0.01	15,096	11,582	3,512	2
	≥0.04	9,981	7,803	2,177	1
	≥0.09	6,300	4,948	1,351	1
	≥0.16	3,812	2,962	849	1
JTI-Small_Intestine_Terminal_Ileum	≥0.01	9,420	7,048	1	2371
	≥0.04	7,119	5,277	1,841	1
	≥0.09	3,853	2,814	1,038	1
	≥0.16	1976	1407	568	1

Protein, Protein coding genes; lncRNAs, long non-coding RNAs; miRNAs, microRNAs.
