# Supplementary material for: Joint analysis of proteome, transcriptome, and multi-trait analysis to identify novel Parkinson’s disease risk genes
Source: Aging (Albany NY). 2024 Jan 17;16(2):1555–80. doi: 10.18632/aging.205444 (PMC10866412; doi:10.18632/aging.205444)
Supplement: Supplementary Table 25 [file aging-16-205444-s024.pdf]

Supplementary Table 25. Characteristics of discovery (ROS/MAP) proteomic dataset.

	Female N	Male N	Age at death	pQTL N	Weights N
ROSMAP	262	114	89.4(6.4)	8356	1475
Banner	87	65	85.5 (7.1)	8168	1139
